# Case of Poisoning from Arsenic

**Published:** 1852-07

**Authors:** A. M. Leonard

**Affiliations:** Lockport. N. Y.


### ART. II. — *Case of Poisoning from Arsenic.* By A. M. LEONARD, M. D., Lockport, N. Y.

I send you the following report for insertion in your Journal, should it in your judgment possess importance enough to give it a place there:

Tuesday, April 13th, 1852, I was called upon to examine the body of

Alexander Brown, who had died twenty hours before, under suspicious circumstances. In company with Drs. Worcester and May, I proceeded to the examination. The surface of the body presented no unnatural appearance, except a marked depression of the right side of the chest. On opening into the cavity of the chest, found evidences of extensive previous pleuro-pneumonitis of the right side, with very extensive adhesions of the lung to the walls of the chest. The stomach presented no unnatural appearance on its peritoneal surface, neither did the bowels, except the colon, which seemed very much contracted. I removed the stomach with the duodenum and the lower two-thirds of the œsophagus, and placed it in an open mouthed jar. Found several ulcers on the mucous surface of the duodenum. On making an opening into the ilium there issued nearly a pint of muco-purulent matter. Liver much enlarged, with its surface granulated, but no signs of recent inflammation in any of the viscera. As it was suspected that he had taken poison, I proceeded, the next day, to examine the stomach for arsenic. On opening into it I found from twelve to sixteen ounces of undigested food, with about the same quantity of a slightly sanguineous fluid. Appearances of inflammation in the mucous surface very slight, but the whole inner wall of the stomach was studded with small clots of blood underneath the villous coat. I also discovered, floating in mucus, and attached to the coats of the stomach, a white powder.

I poured the contents of the stomach into a vessel, and boiled them for fifteen minutes, when, in company with Dr. Worcester, I proceeded to test the fluid by the arseniated hydrogen test. Our apparatus was simply a Florence flask, with a cork nicely adjusted, through which passed a glass tube, furnished at its upper end with a jet of fine bore.

We first tested our reagents, when we got nothing but the characteristic flame of hydrogen, producing no stain whatever upon glass or porcelain. We then introduced ℥ss. of the fluid from the stomach, applied a lighted taper to the gas as it came over, when we at once got the dull white flame of arsenic. A plate of porcelain was brought over the flame, when it was immediately coated with a thick, metallic crust of arsenic, and so abundant was it that we have no hesitation in saying that, from one-half ounce of fluid we might have coated a surface of eighteen inches square with metallic arsenic. Not wishing to inhale the black suffocating smoke which it so abundantly produced, we were obliged to throw up the windows and let it pass off.

This experiment, of itself, was perfectly satisfactory to us, though hastily performed, and not with that precision which would be desirable inasmuch as we were to testify in a court of justice. The wife of the diseased, with several domestics were under arrest, and the court was waiting for us; yet, in a hurried manner, we instituted several other tests.

We removed from the coat of the stomach from six to eight ounces of the white powder, made an aqueous solusion of it which saved us the necessity of filtering. Upon a portion of this fluid we instituted the ammoniacal nitrate of silver test, and readily produced the characteristic lemon yellow precipitate, which soon passed to a dark brown, on exposure to the light.

We next proceeded with the ammoniacal sulphate of copper test in the same manner, and readily produced the grass green precipitate. We burned a portion of the powder as obtained from the stomach, upon charcoal, and obtained a distinct garlic odor. We readily obtained the metallic stain and the arsenious acid by holding a glass tube over the flame in the hydrogen test. And so of every test we instituted, they all gave the evidence of arsenic in a large quantity.

The following facts were elicited on the examination:

He had been a very intemperate man, had lived very unhappily with his wife, and for the last two years had been much out of health. On the morning of his death he went down into the town, a distance of three quarters of a mile, and immediately on his return he was seen to drink a fluid from a saucer, upon the surface of which floated a white powder. He soon commenced vomiting, complained of pain in his stomach and bowels, and of feeling faint. There was more or less blood thrown up with the contents of his stomach. At the end of two hours a homœopathic physician was called in and there was therefore nothing done for the patient.* He soon sunk into a collapsed state, and died in five hours after the taking the poison. Some three hours after taking the poison he had several dejections, which were described as being of a strange appearance, and from this description we should think they partook very much of the character of the muco-purulent matter we found in the bowels.

It is a query in our minds whether the poisoning could have had any thing to do with producing that condition of the bowels.

We have no means by which to estimate the quantity taken. But judging from that which remained in the stomach undissolved, he must have taken a drachm at least. It was the large quantity which destroyed life so speedily, we have no doubt, and his speedy death accounts for the want of inflammatory symptoms.

---

* Would the Homœopathist in such a case give, as a remedy, an infinitessimal quantity of arsenic, in lieu of the hydrated per oxide of iron ? — Editor.

